# Healthy Trust and Pandemic Readiness in the Context of Messenger RNA Vaccines: Protocol for a Sequential Deliberative Dialogue Study

**DOI:** 10.2196/91534

**Published:** 2026-09-25

**Authors:** Katrina Plamondon, Rodrigo Curty Pereira, Angeli Rawat

**Affiliations:** 1 School of Nursing, Faculty of Health and Social Development, University of British Columbia, Okanagan Campus, Landmark 4 Bldg, 6th Fl, 1628 Dickson Avenue, Kelowna, BC, BC, V1Y 5Y7, Canada, 1 250-807-8681

**Keywords:** deliberative dialogues, pandemic readiness, mRNA vaccines, immunization, trust, vaccine hesitancy

## Abstract

**Background:**

Pandemics threaten economies, security, and public health, requiring strong global and local collaboration and coordination. Public receptivity to emergent vaccine technologies is essential to pandemic readiness. Although messenger RNA (mRNA) vaccines offer rapid adaptability and hold promise, their use during the COVID-19 pandemic was met with polarizing debates that raise questions about the conditions needed to support acceptable and equitable use of new vaccines.

**Objective:**

This deliberative dialogue study aims to generate consensus on how to ensure informed, accessible public engagement and strengthen public trust in a climate of divisiveness and misinformation, disinformation, and malinformation. Using mRNA vaccines as a case study, the project’s specific research objective is to seek insights into what people believe is needed for public understanding, equitable access, and acceptance of emerging vaccine technologies.

**Methods:**

Using a sequential deliberative dialogue design, this study invites individuals with significant influence on pandemic readiness and those most likely to be disproportionately affected by related decisions into reflective, conversational, workshop-style online events and self-led dialogues. Dialogues and guiding questions are informed by a knowledge synthesis on what is unknown about mRNA vaccine public sentiments based on a rapid review, an environmental scan, consultations with experts across sectors, and ongoing updates from the dialogues. Data will be analyzed in situ in an iterative and collaborative synthesis of general agreements between dialogue contributors in response to guiding questions and emerging themes. Dialogue reports will be drafted by the research team based on observational notes, transcripts, and video recordings and reviewed by contributors. In summative dialogues, the research team will present main threads of dialogue, responding to research objectives and newly identified knowledge gaps and collecting input to inform dialogue outputs.

**Results:**

This study was funded in May 2025. As of June 2026, we have conducted 10 deliberative dialogues and supported 1 self-led dialogue, engaging 77 contributors across sectors and disciplines. We held 2 summative dialogues with 18 attendees, where we presented the main threads of dialogue as identified by the research team through collaborative analysis. We have started incorporating input from closing dialogues into the final reports and expect to publish the results in the second half of 2026, both as lay-language reports and peer-reviewed manuscripts.

**Conclusions:**

This protocol outlines a consensus-building process capable of deepening the understanding of vaccine hesitancy and strengthening pandemic readiness and public trust in Canada. Dialogues are expected to provide general agreements on how to enhance science and health literacy, tailor communications considering historical inequities, respond to the needs of health care workers, and navigate polarization around vaccination. By creating space for shared learning and collective insight, this study supports the rebuilding of trust in public health institutions as they adapt to evolving societal expectations.

## Introduction

### Pandemic Preparedness, Public Trust, and Vaccine Receptivity

Pandemics pose critical threats to economies, security, and public health around the world, demanding intense global and local collaboration and coordination. While “pandemics begin suddenly and never really end,” viruses involved in pandemics tend to linger for generations [1]. The influenza virus in what is commonly known as the 1918 “Spanish flu” pandemic became an ancestor or “founder strain” of an enduring pandemic era that continues today [2]. The first quarter of the 21st century was marked by 8 significant outbreaks, 2 of which were declared pandemics. Catalyzed and perpetuated by human attitudes and actions, pandemics are inevitable, massive disruptions to human health and sustainability [1,3,4]. Trust in governments, public institutions (eg, health systems), and science (and how it is extended, earned, or withdrawn [5]) plays a pivotal role in social readiness to navigate such catastrophic public health crises. The likelihood, scale, and impacts of future pandemics will be determined, at least in part, by the degree of basic trust within and between societies. Public engagement and trust are among many dynamic factors influencing how viruses emerge, move through, and affect populations. In the context of pandemic readiness, given that vaccines are essential medicines and means of preventing or containing the spread or severity of disease, public receptivity to the use of emergent therapeutic technologies in vaccines is particularly poignant.

Vaccination programs are public goods, meaning they are nonrivalrous, meaning one’s use does not prevent others’ use, and nonexcludable, meaning one’s enjoyment of the benefits of the good generally does not exclude others. Herd immunity resulting from vaccination programs demonstrates this concept, providing health-protecting benefits by containing and decreasing health consequences, extending beyond those individuals who receive vaccines [6,7]; they have the potential to benefit all people, everywhere, whether or not people directly contribute to eradication. Vaccines themselves are not necessarily public goods because they involve intellectual property, corporations, and competing national interests that all shape access to these essential medicines [8,9]. Tensions between the public good of vaccine programs and the private benefits of vaccines themselves play a role in shaping public sentiments about the beneficence of those involved in making decisions about public health restrictions or recommendations. Vaccines and vaccination programs are both critical ingredients in protecting the collective, global capacity to endure future pandemics. Access to and acceptability of vaccines, their global distribution, dependency on private corporations in their development, use of therapeutic innovations, and the individual or collective obligations to others are among the many complex ethical and moral debates that emerged during the COVID-19 pandemic [6,9,10]. The use of messenger RNA (mRNA) as a therapeutic innovation (ie, the delivery of synthetic mRNA that prompts an immune response by instructing production of a specific protein, often viral antigens, without requiring direct exposure to the actual virus) in vaccines is promising, given its rapid adaptability. Its use, however, has sparked rigorous, sometimes polarizing, tensions and debates [11,12] that pose critical questions for pandemic readiness.

Research and vaccine development using mRNA technology enabled a rapid response to a new pathogen, COVID-19, early in the pandemic [13-15]. As a promising rapid, adaptive technology that can be used to create vaccines for emergent pathogens, mRNA technology itself can also be considered a public good [16-20]. Global cooperation in the review, coordination, and conduct of clinical trials also contributed to an unprecedented progression along the immune-engineering and biomanufacturing pipeline, leading to a short timeline from vaccine discovery to delivery [13,15]. Despite decades of scientific advancements in the use of mRNA in a wide range of therapeutics, this technology was unfamiliar to the public at the time COVID vaccines became requirements for travel or access to public spaces [12]. As vaccines became available, public dialogue about vaccine preferences, manufacturers, and technology was also unprecedented. Controversy and divisiveness about a wide range of vaccine-related topics emerged, spanning rights [21], entitlements and issues of equity in access [22,23], scientific process and ethics [19,24,25], brand preferences [26], safety [25], profit and trade-related aspects of essential medicines [27,28], and political motivations [12,29]. Debate included deep skepticism from within the scientific community [30] and ranged so far as to include the purpose of mRNA technology itself, with skepticism and conspiracy theories dominating some of the public discourse [31-34]. As this debate unfolded, the public was also flooded with data, without tools or capabilities for interpretation, while also becoming entangled in growing waves of misinformation and disinformation associated with rising populism, polarization, and shifts in the pervasiveness of AI.

Layered within this perfect storm of mutually reinforcing and propelling factors was a mix of communications failures. The media became a primary source of information for the public, fueling worry, uncertainty, prejudice, and divisiveness, while also providing conflicting or inaccurate information about prevention measures or cures, making the media a critical, yet vulnerable, communications platform [35,36]. Political and public scrutiny and politician-led misinformation [37,38], compounded by collective desperation, frustration, and anger in response to pandemic-related public health measures, often overshadowed the voice, practice, and leadership of public health officials and providers. Public health officials’ trust in people’s autonomy and informed decision-making was challenged, and vice versa [39]. Overall, communications failed [37,39-43]. Responsiveness to emerging evidence, reflected in shifts in public health messaging over time and complicated by an “infodemic” [36,44], was frequently met with skepticism about public health competence, scientific integrity, government interference, or general beneficence of people involved in any aspect of developing, regulating, manufacturing, or delivering vaccines. Public health communications were inconsistent across jurisdictions and often met with public pushback and political tensions. Trust in vaccines, their safety, necessity, and intent, broadly diminished [23,24,43,45], while, simultaneously, pandemic-related interruptions to routine public health programming affected vaccine coverage across Canada and around the world [46-49]. Post pandemic, we are now witnessing new outbreaks of vaccine-preventable diseases [50-54] while anticipating significant and avoidable risks to collective preparedness and capacity to respond to future pandemics. The example of trust and communications related to mRNA technology, as a foundational ingredient for adaptive and responsive vaccines, presents a particularly poignant opportunity to learn, articulate normative expectations, and build consensus on the communication standards and public health leadership necessary to protect public interests and collective, global, and population health.

In this research, our understanding of trust is based on nuanced discussion of trust by Fox et al [5], which proposes that public trust in a broad range of institutions and authorities can be conceptualized as a continuum with extreme distrust on the one end and unquestioning trust on the other. The continuum describes six distinguishable degrees of trust:

1. Distrust: An absence of trust characterized by cynicism and suspicion.
2. Mistrust: A tenuous kind of trust, characterized by skepticism, particularly in the motivations and intentions of what the authors refer to as “elite actors”.
3. Informed trust: Trust earned by virtue of active verification through seeking, reviewing, and/or interpreting evidence.
4. Basic trust: Tacit, earned through repeated positive experiences with government or other elite actors.
5. Uninformed trust: Unearned trust reliant on deference to social pressure or persuasion from people who are perceived to be more informed or knowledgeable, which can include trust extended unquestioningly to health care providers or to bodies of information or evidence.
6. Unquestioning trust: Faith-driven or fundamentalist extensions of unearned trust or overconfidence in absolutes, including the absolute ability of powerholders, or science, to be correct or to act in others’ best interests.

Many factors can influence the overall “bank” of trust existing in society at any given moment as they pull or push people in different directions along this continuum [5]. The overall degree of public trust in something directly affects degrees of public compliance with recommendations, measures, or mandates issued to contain or prevent the spread of disease.

Recognizing the complex interplay of these distinct nodes of trust, and the necessity for rapid development and deployment of vaccines in the face of lingering or emerging pandemics, the main objective of this deliberative dialogue study is to generate consensus on how to ensure informed, accessible public engagement and to strengthen public trust in a climate of divisiveness and misinformation, disinformation, and malinformation. Herein, misinformation is understood as inaccurate information often shared on social media, disinformation refers to the intentional creation and dissemination of false or deceptive information, and malinformation stems from evidence but is exaggerated or misinterpreted [55-57]. The project’s specific research objective is to seek insights into what people believe is needed for public understanding, equitable access, and social acceptance of emerging vaccine technologies using mRNA vaccines as an example.

We adopt a responsive and emergent study design in 2 waves, with the second designed in direct response to what emerges from the first. The first wave of dialogues focused on learning from the COVID-19 pandemic and subsequent outbreaks of known and emerging viruses, such as the current H1N1 avian influenza and measles. With its potential to rapidly adapt to emergent viruses, and as a precursor to emergent therapeutics, such as self-amplifying RNA, this wave of dialogue will focus on public engagement and its relationship to public trust and receptivity to the use of mRNA technology in vaccines.

### Bridge Research Consortium

The protocol presented here is situated within the Bridge Research Consortium (BRC) as one branch of its engagement across settings and sectors. The BRC is a Canadian research initiative hosted within Canada’s Immuno-Engineering and Biomanufacturing Hub (CIEBH) that brings together leading researchers from across the country to study and support public trust in, and equitable access to, vaccines and other immune-based health innovations. It bridges the life and bioengineering sciences with public health, the social sciences, and the humanities to better understand diverse public perspectives, address misinformation and disinformation, enhance vaccine confidence, and promote health equity. The consortium uses mixed methods and engages in participatory action research and dialogue with communities, policymakers, industry, and scientists to cocreate knowledge and solutions that strengthen biomanufacturing and immunization systems in Canada.

## Methods

### Deliberative Dialogue Approach

Deliberative dialogue is a relational method suitable for building consensus by providing an opportunity and strengthening the capacity to weigh the complex interplay of perspectives, ethics, evidence, theory, and practices involved in an issue of shared interest [58]. In this study, deliberative dialogue serves as a coproduction [59,60] and a systematic learning [61] effort to bring people with diverse backgrounds together for evidence-informed, reflective, and appreciative consensus-building. Dialogues serve as a place for the collaborative articulation of the kinds of futures that people want to see unfolding (in this case, relative to pandemic preparedness). This approach aligns with our commitment to integrating equity considerations in and through research, recognizing that equity implications are always present in policy or practice directions relative to public goods [62].

**Figure 1.**
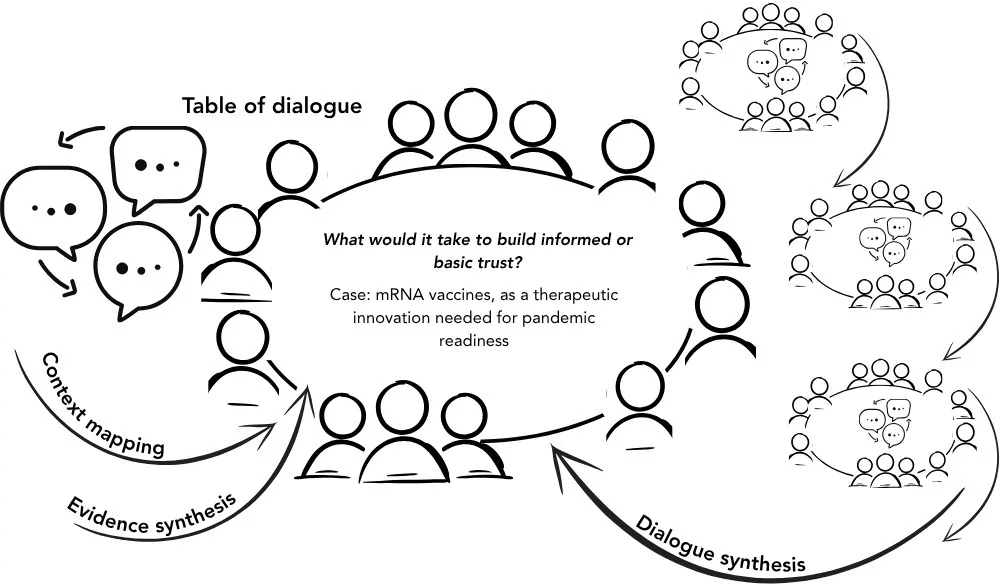
Inviting many perspectives and voices to a broad table of dialogue. mRNA: messenger RNA.

We draw from the critical pedagogy by Freire [63,64], with its attentiveness to the possibilities that exist in using dialogue to learn from each other in ways that collectively construct equity-promoting pathways toward a shared future. This philosophical foundation supports our use of relational approaches to explore pluralistic equity, ethics, and practice implications for pandemic preparedness and response. In this case, we can imagine each interaction with participants as an invitation to join a “table of dialogue” (Figure 1).

For the duration of the dialogue, people who join the table are guests who are offered a carefully curated set of opportunities to engage with each other around a specific case, idea, or issue. Each “course” presents purposeful questions and activities designed to invite them into sharing reactions, insights, and interpretations toward deepened understanding and appreciation of others. As they hear from others at the table, they learn about and examine a particular case or idea in ways that are situated in context and therefore reflective of broader sociopolitical ecosystems.

### Study Design

This deliberative dialogue study will engage different groups of people who have some form of connection or interest in the use of mRNA therapeutics during pandemics (described below). It is modeled after the rigorous dialogue-based research underpinning the elaboration of the Canadian Coalition for Global Health Research (CCGHR) Principles. Deliberative dialogue involves bringing a purposively identified group of people together in structured conversations that respond to a knowledge synthesis [58,65,66]. Given the desire for broad engagement on a topic of high relevance across society, we will use a sequential dialogue design. In this design, cycles of synthesizing and engaging iteratively progress toward a desired outcome while extending the number of people (and thereby diversity of perspectives) engaged [58,65]. Sequential dialogues also enhance the trustworthiness of the findings (especially credibility and dependability) by offering several member-checking opportunities to contributors [67], who are welcome to take part in as many dialogues as they wish, provide input on the dialogue outputs, and participate in summative dialogues.

**Figure 2.**
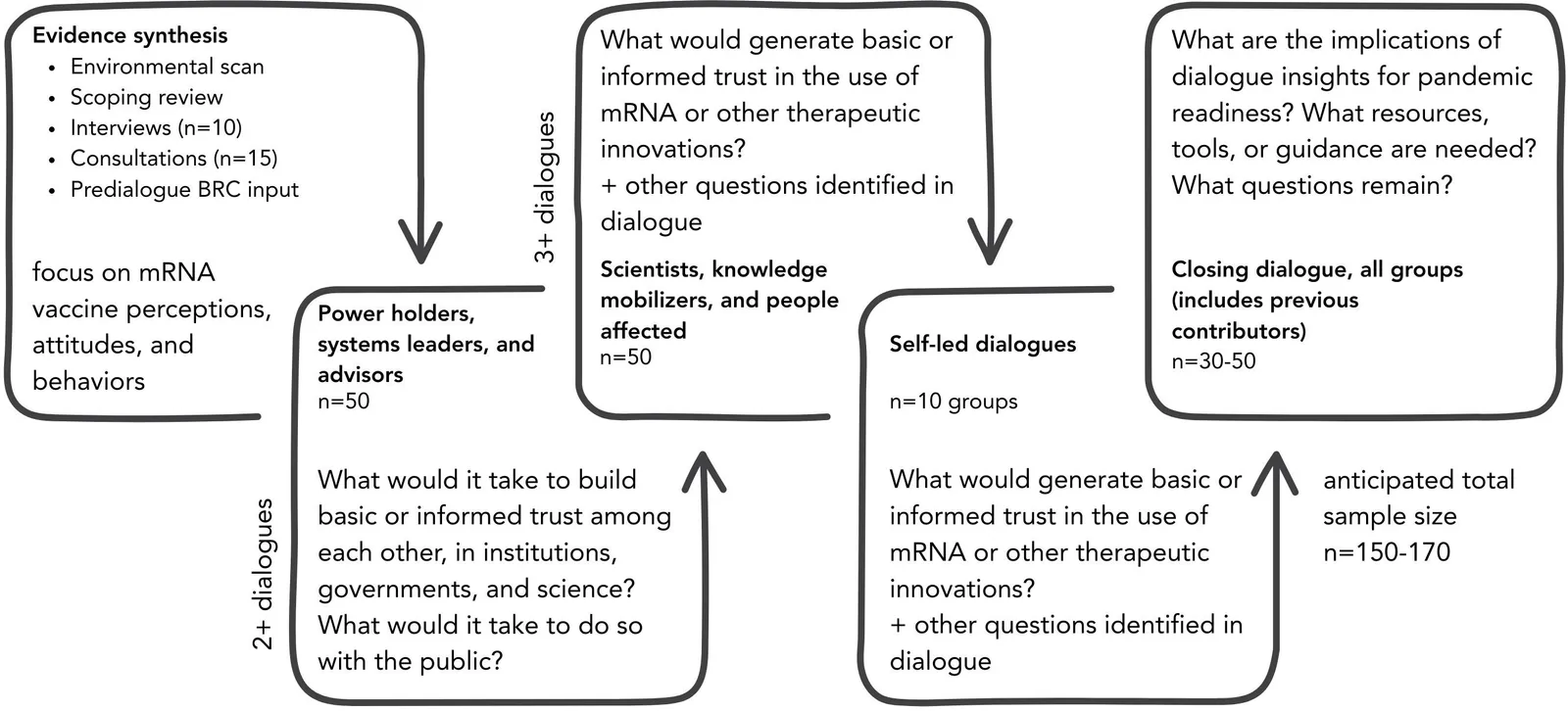
Sequence of dialogue, focused on the use of mRNA (messenger RNA) therapeutics in vaccines. BRC: Bridge Research Consortium.

The sequential series will involve a beginning set of dialogues followed by targeted, facilitated dialogues, invitations for self-led dialogues using a dialogue toolkit, and closing summative dialogues (Figure 2). This design provides a foundation for validating and refining the evidence syntheses prior to engaging specific groups. It also allows a diversity of perspectives to contribute to consolidating calls for action, recommendations, and dialogue-driven outcomes or outputs. Further, it is a responsive and adaptive design, wherein each phase of engagement and synthesis informs subsequent dialogues. While each dialogue will be inevitably shaped by the people and perspectives at that particular “table,” their insights, interpretations, and recommendations will be synthesized and shared alongside the evidence summary at subsequent dialogues.

### Preparing for Dialogues: Synthesis and Consultation

Synthesis activities informing the first wave of dialogues included a rapid review of the literature on public perceptions and responses to the use of mRNA technology in vaccines, an environmental scan of public health communications relative to the use of mRNA technology in vaccines, interviews, and the integration of emerging findings from relevant BRC studies (Table 1). These syntheses will serve as the foundational content for the development of materials (eg, predialogue briefs and facilitation slides) that will inform and guide the dialogues. For the second wave of dialogue, we will follow direction given by contributors to the first wave to conduct responsive evidence reviews (as needed), integrate relevant research results emerging from BRC, and adapt to any changes in context.

**Table 1.** Summary of predialogue synthesis and consultation activities.

Synthesis activity	Purpose and guiding question	Planned outputs
Scoping review of peer-reviewed literature	Identify empirical evidence on the relationship between attitudes, receptivity, reluctance-skepticism-refusal, and general perceptions of the use of mRNA technology in essential medicines (eg, vaccines) to inform dialogue materials. “What do we know about perceptions, attitudes, and behaviors around the use of mRNA^a^ technology in therapeutics?”	Manuscript to submit for peer review and publication Predialogue briefs and infographics
Environmental scan, review of peer-reviewed and gray literature	Provide foundational overview of what is available from public policy and practice leadership arenas from points of intersect between the public and service providers, where mRNA technology (eg, vaccines) would be administered. “What do we know about the discourse and decision-making around mRNA and emerging immuno-technologies within government, health authority, and health care organization ecosystems (challenges and barriers)?”	Manuscript to submit for peer review and publication Predialogue briefs and infographics
Meetings and consultation	Listen to the perspectives, wishes, and hopes of people within the BRC^b^ who have a vested interest in the study topic. Enhance review efforts, providing insights on mRNA contexts and relevant work, informing development of deliberative dialogue scope, focus, and design. “What questions or points of tension would you want this series of dialogues to explore? Who do you think should be engaged? What outcomes or solutions would you like to see?”	Meeting notes Predialogue briefs
Collaborative context and content analysis	Situate knowledge syntheses efforts in an intensive, applied deliberative dialogue training and planning session. “What deliberative dialogue focus and design (who, what, and how) best respond to the goals of engagement, the evidence and knowledge available to us, and the insights shared by BRC team members and collaborators throughout our synthesis process?”	Context mapping exercise (reflected in the contributor groups named below and in the questions guiding dialogue)

a mRNA: messenger RNA.

b BRC: Bridge Research Consortium.

### Study Setting and Sampling Strategy

Pandemics and other global public health crises, by nature, affect all people everywhere. We situate this study in a Canadian context, with an interest in representation across geographies and populations. To begin, the dialogues will focus on those with a role in public health leadership or practice across Canada, from which we will expand to include other groups. Following an in-depth context and power analysis [68], target audiences for this series of dialogues were narrowed to include people (1) whose perspectives, roles, and spheres of influence position them to play a pivotal role in pandemic readiness or (2) who are most likely to be disproportionately affected by the decisions or actions of those in pivotal roles, relative to pandemic readiness (Table 2).

**Table 2.** Summary of contributors by group.

Contributor group	Who are they?
Power holders (individual or group) in positions of authority or influence over the distribution of resources, including information	Government officials in senior roles (eg, ministers or deputy ministers; elected officials) Industry leaders or representatives who shape engagement and/or access policies or practices in private sector settings Media professionals (eg, reporters and social media influencers) engaged in vaccine and biotechnology communications Editorial networks or groups that set practice standards (eg, integrity practices for preprints and peer-review standards) Accreditation or other professional bodies involved in setting standards, expectations, or consensus guidelines Others who hold decision-making or resource-allocation power
Systems leaders in positions to set public policy directions, issue public mandates, or design implementation strategies	Public health leaders and providers, in official roles at local, provincial or territorial, or national levels Health systems leaders in roles related to professional practice Others in systems leadership roles
Scientists and knowledge mobilizers with training and expertise related to a range of topics	Researchers in: Discovery, monitoring, and evaluation Health surveillance Public health, health promotion, and protection Epidemiology and population health Ethics Health communications relevant to pandemics Others as identified through dialogue
Advisors who do not currently occupy an official office but offer insights relative to their experience or connections to governance bodies	Professional associations Accreditation bodies Indigenous Nations’ leadership or advisory groups Others as identified through dialogue
People affected by or involved in either delivering services related to vaccines, under the mandates created by others, advising on or making decisions about receiving messenger RNA (mRNA) therapeutics	Licensed health care providers either engaged in communications related to vaccines, or who were vaccine-hesitant, including direct care providers in acute and community care (eg, nurses; primary care providers such as physicians or nurse practitioners; and community pharmacists) Members of the public who experience heightened challenges relative to trust or conditions that preclude representativeness in clinical trials (eg, those often underrepresented in research sampling practices, involved in patient voices networks, parents or guardians who make decisions about immunizations for dependents)

We will invest effort in recruiting contributors from each of the subpopulations of interest (Table 2). However, we anticipate that the recruitment strategy will be more successful among some groups than others depending on existing connections, interest, and availability. More specifically, contributors will include public health leaders, policymakers, health professionals, direct care providers, journalists, people with lived experiences (eg, long COVID, immunocompromised groups), leaders of civil society organizations, scientists from across the bio-manufacturing and immuno-engineering and communications, knowledge mobilization, social, population, and public health sciences. In the face of power asymmetries between these groups, the dialogic methodology provides a useful approach where contributors are invited to participate as their whole selves in an informal conversation where diverging opinions and levels of expertise are promoted rather than judged negatively. Dialogue facilitators are trained to identify varying levels of engagement and invite participation in ways that are most comfortable to contributors (eg, orally, via Zoom chat or on the whiteboard). Finally, part of the predialogue preparation entails learning more about expected contributors, which equips facilitators to invite contribution on topics with which participants feel familiar.

At the end of each dialogue, we will return to our sampling goals in the context of what we hear through dialogues and adjust our recruiting strategies in response. We will invite anyone who participated in a key informant interview or a dialogue and extend invitations through the BRC and its networks. As we extend specific invitations to contribute to dialogue, we will seek broad geographic representation from across Canada and use strategies to optimize the diversity and representativeness of contributors. Should contributors identify additional audiences or groups for inclusion as dialogues unfold, we will consider adding more dialogues as we are able.

Target sample size decisions for this sequential dialogue study are guided by the guidance of Malterud et al [69] on factors influencing information power and sampling needs in qualitative research (Multimedia Appendix 1). We targeted a more extensive and larger sample size to ensure strong information power, given the characteristics of the study: (1) broad aim, including articulating normative standards or consensus statements; (2) highly variable experiences, knowledge, and properties among people who are contributing to dialogue (ie, sparse specificity); (3) broad theoretical foundation that informs rather than directly applies or constricts dialogue; (4) rich dialogue, with strong and clear focus on coconstructing insights and interactions and highly skilled facilitation; and (5) cross-case analysis strategy, enabled through our iterative processes of synthesizing and engaging.

We anticipate approximately 15 to 30 participants per dialogue and an overall number of contributors of approximately 150 to 170 people (Figure 2). This estimate excludes those who contribute to self-led dialogues, which we anticipate will involve approximately 10 submissions. We will monitor and assess information power as the dialogues unfold.

### Inclusion and Exclusion Criteria

Participants in dialogues must be (1) purposefully invited into dialogue by virtue of an environmental scan whose expertise, life experience, critical reflection, and spheres of influence position them to both contribute meaningfully to, and/or act on, vaccine equity, accessibility, and acceptability in Canada; (2) 18 years of age or older; (3) fluent in English; and (4) able to attend the dialogue in the format offered (in person or virtual).

Respecting the risks that have come to be associated with leading vaccine-related research [70,71] and because of the social positionalities of our research team members (ie, a gender-diverse and multiethnic group), we will exclude groups or individuals with strong antivaccine or antisocial sentiments from virtual or in-person dialogues; however, self-led dialogue remains an option available to such groups.

### Recruitment

Participants in the dialogues will be identified from a recruitment planning process that considers their public contributions to issues of immune-based innovations (eg, vaccine equity, accessibility, and acceptability in Canada). Using existing email networks through the BRC and publicly available email addresses, participants will be contacted by the study team using the Equity Science Lab UBC (University of British Columbia) email address (equityscience.lab@ubc.ca), provided with information about the study, and invited to sign up to participate in a dialogue. They will be invited to pass the invitation along to others whose perspectives or insights would be ideal as contributors to dialogue. The snowball recruitment strategy is justified for the action-oriented design of the research, where we seek to invite people capable of shaping pandemic preparedness and who have direct insight into public health decision-making. Although we expect general common interest in talking about mRNA vaccines, many contributors tend to operate in silos, thus not having the opportunity to be in dialogue with people across sectors and population groups. The dialogues are, therefore, an opportunity to bridge these groups.

Recognizing that the use of snowball recruitment poses a risk of amplifying existing networks and excluding marginalized perspectives, we will encourage contributors to identify groups that should be included in dialogues. With the exception of groups that may pose risks to the research team [70,71], we will make efforts to include those perspectives, including youth, people with lived experiences relevant to mRNA vaccines, civil society organization leaders, and health care workers in direct contact with patients with varying vaccine sentiments. Additionally, the study will be advertised through public events (ie, webinars, workshops at public health conferences, and the public BRC website), where audiences are given the option to sign up for dialogues or request a self-led dialogue toolkit. The dialogues thus far revealed diverse attitudes toward public health, governments, and science communications, including groups that are skeptical of government officials or with a history of medical negligence.

### Dialogue Process and Formats

#### Procedures and Implementation

Participants will receive a lay-language synthesis of the scoping review and ongoing research results before each dialogue. Dialogues will use a variety of participatory workshop-style techniques to deliberate on the implications of research findings, with each activity focused on iteratively reaching consensus. Consensus-building methods such as deliberative dialogues do not require all contributors to think the same way or to reach a singular unified course of action. Instead, they invite perspective-taking to coproduce evidence-informed solutions that support forward movement with general agreement [72]. Dialogues will be held mainly online using Zoom but also in person at opportunistic events (eg, alongside a relevant conference). At each dialogue, we will apply the nonattribution principles of the Chatham House Rule [73]. Each dialogue event will be reported on as a series, with an evolving synthesis updated at the end of the first wave of dialogue. Invitations will also be extended to groups interested in holding a reflective conversation independently, with a Qualtrics submission form for them to offer their input using the same dialogue brief and question prompts guiding facilitated dialogues. A second sequence of dialogues will be planned in late 2026, responsive to direction offered by the first sequence and to changing needs or contexts.

In each dialogue event, we will invite direction about dissemination products and resources that would serve the needs of relevant audiences. Some people might choose to participate in more than one way by, for example, joining a facilitated dialogue and then using the dialogue toolkit to host a local dialogue within their own organization.

#### Predialogue Engagement

One week before a scheduled dialogue, each participant will receive an email reminder, a request to complete a perspectives and demographics survey, and a predialogue information package. The predialogue information package will include a 1-page summary of dialogue purpose, evidence-synthesis highlights (with links available for people to access full reports, should they wish to do so), and questions for reflection. The demographic-perspectives survey will gather information on age range, gender, ethnocultural background, preferred languages, career stage, previous equity-related training, disciplinary and geographic perspectives, and other relevant perspectives. This demographic information will inform sampling, recruitment efforts, and potential intersectionality-informed analysis.

#### In-Dialogue Engagement

For both dialogues hosted online and in-person, dialogue spaces will be open 30 minutes in advance to offer people a casual and warm welcome to the dialogic space. Facilitators will guide contributors through a progression of large and small-group reflective conversations. At the beginning of the dialogue, the lead facilitator will provide a brief overview of the event and highlight consent procedures for nonattribution. After introductions, a presentation about the purpose of the dialogue will follow, with highlights of the evidence syntheses and insights from previous dialogues. Following this presentation, engagement in the dialogue itself will be like participating in a lively, interactive workshop responding to broad, open questions designed to spark dialogue.

The lead facilitator will use reflective summarizing, probing questions, and reframing strategies to support a deeply reflective and rich dialogue. For in-person dialogues, contributors will also have an opportunity to contribute to the collective recording of dialogue highlights through writing or drawing on mural boards. Question prompts for each dialogue will be reviewed 3 days before the event, taking into careful consideration any guidance or direction offered from previous iterations of dialogue or questions arising from emerging results. The final 20 to 30 minutes of each dialogue will be reserved for closing reflections and commitments, with 3 to 5 minutes dedicated to providing information about what contributors can expect postdialogue and what opportunities are available for continued engagement. Each dialogue will be recorded (audio and video, when possible), with note takers recording key insights and highlights in a note-taking guide (Multimedia Appendix 2).

#### Postdialogue

Within 2 weeks of a dialogue, contributors will be provided with a draft report of dialogue and a link to access a dialogue toolkit. We will invite contributors to decide if they wish to be acknowledged as a contributor. If they wish to participate in a review of the report, they will have 1 to 2 weeks to provide comments or clarifications. Once all feedback on the report of dialogue is received, the facilitation team will integrate the comments and finalize the report.

#### Self-Led Dialogues

For those contributors who choose to access the dialogue toolkit, they will find a set of materials that can be used to support and report back on a 30- to 60-minute conversation among smaller groups. We anticipate approximately 10 groups to hold these brief report-back dialogues (2 per interest group in Table 2). Reporting back on these dialogues will be enabled through a Qualtrics survey link, provided as a digital link and a QR code with the toolkit. The toolkit will include a brief overview of the same content presented at the beginning of facilitated dialogues and is designed to support integrating dialogue into routine meetings or adapted for asynchronous contributions.

### Data Handling and Analysis

Dialogic studies rely on active analysis and synthesis in situ, or while the dialogue unfolds, through the iterative generation and critical consideration of data that are collective, reflecting multiple perspectives, and shaped by interpretive acts. Data will be analyzed using procedures for qualitative data, maintaining the interpretive nature of data generated in dialogue by balancing categorizing and contextualizing strategies [65]. Sources of data may be reviewed multiple times if there are further conversations within the team on the perspectives being shared. These pieces are analyzed holistically, whereby verbal and nonverbal data [74] are interpreted together to deepen description and interpretation of meaning. During review of the audio or video data (alongside experiences of the facilitation team), the research team will take particular note of the interaction of verbal and nonverbal communication, which allows us as researchers to (1) corroborate speech narrative (ie, triangulation); (2) capture underlying messages (ie, complementarity); (3) discover nonverbal behaviors that contradict the verbal communication (ie, initiation); (4) broaden the scope of the understanding (ie, expansion); and (5) create new directions based on additional insights (ie, development) [74].

In each dialogue, at least 3 research team members will be engaged in concerted data generation and analysis. While the facilitator leads the dialogue and oral synthesis, the other 2 members take notes following a template adapted to each dialogue, which contains fields for general comments, guiding questions, emerging themes, and key closing comments (Multimedia Appendix 2).

At the end of dialogues, when contributors leave the Zoom meeting, the research team will discuss general impressions and draft the main themes for the dialogue report. Note-takers and the facilitator upload their notes to a shared online folder alongside the recordings and transcripts. One team member is responsible for drafting a report synthesizing the main themes of dialogue and using illustrative quotes, which is reviewed by other team members and edited accordingly. This report is then shared with dialogue contributors for member-checking [75]. Inputs from contributors are included in the final version of dialogue reports.

At the end of the dialogue series, the principal investigator will identify the main threads of dialogue, that is, the main deductive and inductive themes recurring through the series. These threads of dialogue will be used as columns in an Excel sheet, and each dialogue report will make up the rows. In pairs, the research team will extract data from dialogue reports relevant to each column. In a peer-review process, the first reviewer will be responsible for initial extraction, and the second will be responsible for member-checking and complementation (eg, adding other relevant fragments, transferring content to other columns). Finally, the research team collectively outlines the summative dialogue reports where each thread of dialogue informs a heading, and the content is a summary of the data in each column. Past dialogue contributors are then invited to participate in summative dialogues, where analysis results are presented and inputs are taken to inform the final version of the reports and other identified knowledge translation products (eg, timelines, policy briefs, and guidelines for relevant decision-makers).

### Ethical Considerations

The study has undergone ethical review by the UBC Behavioural Research Ethics Board (REB H25-00870). If and when adjustments are needed, we will submit postapproval activities to this ethics board. For privacy and confidentiality, this study adopts a nonattribution principle, meaning that we invite people to listen and learn from each other during the dialogue and to share what they learn with others after the dialogue without disclosing the identity or words of anyone at the event. In all reporting, including dialogue summaries shared only with participants, no names will be associated with content. In addition, we will follow equity practices for meaningful inclusion [72], with attention to extending warm welcome through all dialogue materials and attention to establishing experiences of welcome during dialogues.

### Dialogue Outputs

We will prepare public-facing reports, which will be available to contributors and others as the dialogues unfold. In addition to the preparation of manuscripts for peer-reviewed publication and knowledge mobilization products, this iterative approach to dialogue will involve moving through periods of engaging and synthesizing, ultimately leading to the possibility of high-impact outputs, such as recommendations specific to improving trust and communications in the context of using mRNA or other emergent technologies in vaccines; products, recommendations, or tools responsive to the direction offered through the dialogues; consensus standards; and recommendations or calls for action.

Participants will contribute toward reimagining the preconditions for healthy trust required for vaccine accessibility and acceptability. Dialogue insights will support future pandemic responses and open broader dialogue on the role of science and trust in science, public engagement, and communication within society. We believe participants will see these dialogues as worthy and important investments of their time and energy and that they will want to have a voice in the process.

Although individuals with antivaccination attitudes are likely to view some of the groups involved in this dialogue with skepticism, there are many other groups within the vaccine hesitancy spectrum whose sentiments are dependent on context, including matters of tailored communication and accessibility [76,77]. This research gathers many groups with decision-making power who can learn from the dialogue itself and from the opportunity to converse with affected populations, journalists, and researchers with diverse backgrounds. These efforts can better inform initiatives targeted at that portion of the vaccine-hesitant population whose unique needs require tailored initiatives, such as the South Asian community in the Simon Fraser Valley, which went from having the lowest COVID-19 vaccination rates to one of the highest coverages after local public health authorities made efforts to engage with appropriate language, media, and communicators [78,79].

## Results

### Scoping Review Findings and Study Development

This study was funded in May 2025. As of May 2026, we have completed the scoping review of the literature on mRNA vaccine hesitancy worldwide, which informed the knowledge synthesis predialogue briefs. Results from this review are being prepared for publication, but gaps and tensions identified informed some dialogue-guiding questions (Table 3). Notably, few studies distinguished between hesitancy specific to mRNA vaccines compared to more general vaccine hesitancy, and there was a significant lack of attention to equity considerations. Furthermore, preliminary dialogues with BRC researchers studying online and media discourse highlighted the need to include action-oriented questions targeted at journalists, social media influencers, and knowledge mobilizers. Therefore, some of our dialogue-guiding questions responded to these themes (Table 3).

**Table 3.** Knowledge gaps identified through a scoping review and research partners.

Knowledge gaps	Examples of added guiding question(s)
Distinction between mRNA-specific and general vaccine hesitancy	What pressing points of tension influence acceptability and uptake of mRNA vaccines? Thinking about what affected trust in mRNA^a^ vaccines, what contributed to building trust? What contributed to eroding trust? What did we learn from mRNA vaccines, and efforts to communicate and promote uptake? What did we hear in everyday conversations about mRNA COVID-19 vaccines?
Equity considerations	Considering those affected by long COVID (or others with unique needs), what other factors should be taken into consideration when thinking of actions to rebuild healthy trust in mRNA vaccines and public health? Where does public health belong? What would it mean to expand the definition of public health?
Communications	What do people working in media and communications need from scientists in order to provide quality information on matters like mRNA vaccines? Where do they currently get information from?

a mRNA: messenger RNA.

### Data Collection and Analysis Progress

Between December 2025 and May 2026, we conducted 10 deliberative dialogues (1 in person and 9 online) and supported 1 self-led dialogue, engaging 77 contributors in total. Contributors included scientists involved in a broad range of research related to vaccines, from discovery and development to manufacturing and distribution and public health, philosophy and ethics, sociology, epidemiology, digital health sciences, and business. Contributors also included direct care providers, journalists, public health communication specialists, public health leaders and decision-makers, patient advocacy groups, not-for-profit sector leaders, and industry representatives.

We held 2 summative dialogues with 18 attendees, where we presented the main threads of dialogue as identified by the research team in collaborative analysis. As of June 2026, we have started incorporating inputs from closing dialogues into final reports and expect to publish results later in 2026 both as lay-language reports and tools (eg, interactive timelines and policy briefs) and peer-reviewed manuscripts.

## Discussion

### Anticipated Findings

We anticipate that dialogues will reveal the need to rethink and restructure relationships between science, public health, and public engagement following lessons learned from the mRNA vaccine hesitancy phenomenon during the COVID-19 pandemic. One relevant expected thread of dialogue is that around the ability to communicate evidence around vaccine safety and efficacy clearly. Although scientists had studied mRNA platforms for years, the general public perceived mRNA vaccines as experimental [80], which public health leaders had difficulty communicating given that COVID-19 vaccines represented the first large-scale manufacturing and distribution of this technology [81].

During the final analysis, we will seek to better categorize mRNA-specific vaccine hesitancy through threads of dialogue directly linked to unique characteristics of mRNA technology and the wider context of the COVID-19 pandemic. However, since dialogic data sits in relationship with context, our interpretation and presentation of results will likely speak to both mRNA-specific sentiments and vaccine sentiments more broadly, that is, the particular in relation to the broader context.

Issues of equity and power are also expected to be considered by contributors, especially in relation to how decision-making around vaccine promotion is communicated. Language barriers and digital divides across generations and geographies may influence how messaging was interpreted, who remained hesitant, and where trust in public health measures was demanded without accommodating cultural or language realities, such as among Indigenous and racialized communities who carry intergenerational and institutional trauma toward health systems [78,79,82,83].

In addition, we anticipate dialogues to point to guidelines on how to resolve tensions between government, public health leadership, and frontline health care workers, who were both recipients and deliverers of vaccines [84]. Although overworked, exhausted, traumatized, and working with incomplete information, frontline staff were expected to trust leaders [5], even as guidance shifted on dose intervals, mixing vaccines, and booster requirements. Therefore, directions on how to rethink the relationship between these groups is anticipated.

Dialogues will also likely contribute to guidelines on how to deal with scientific disagreement within pandemic preparedness. During COVID-19, tensions emerged within and between public health communities, including between scholars, researchers, and leaders in public health, where valid criticism or questioning of proposed interventions and treatments was often disregarded or labeled as misinformation by other members of the same group [81].

Finally, we expect contributors to reflect on the association of mRNA vaccine acceptance with political affiliation [85], particularly in the digital space. Social media accelerated polarization between groups with opposing views on vaccines and seemingly associated political agendas, creating environments where people primarily consumed information that reinforced their own beliefs [86]. We anticipate calls to “depoliticize” public health communications and suggestions on how to identify and foster shared values that promote population health across the political spectrum.

### Strengths and Limitations

This protocol outlines a unique study designed to equip researchers and professionals across sectors with ways of working together for pandemic preparedness as a response to the divisiveness and misinformation, disinformation, and malinformation as witnessed during the COVID-19 pandemic. The study itself provides a platform for people who would otherwise have few opportunities to engage with each other, despite their overlapping and often coinfluential roles as consumers, scientists, communicators, practice or policy leaders, care providers, or decision makers involved in some aspect of the immunotherapeutic-related dimensions of pandemic readiness. Our focus on mRNA vaccines offers an illustrative example of how science and therapeutic innovation can get entangled in sociopolitical tensions. Coproduction and dialogic efforts give us the opportunity to prepare for future health emergencies in response to context-specific pressures.

The study is limited in its capacity to engage with individuals and groups with more negative sentiments toward mRNA vaccines and vaccines in general. As outlined in other sections, efforts will be put into recruiting people with diverse perspectives, including those who may have had negative experiences with vaccine promotion and roll-out during past health emergencies. Nonetheless, our study will include researchers and professionals who are better equipped and connected to engage with antivaccination and vaccine-hesitant groups, which gives them the opportunity to test the general agreements achieved in this study and provide feedback to our ongoing community of practice.

### Conclusions

This deliberative dialogue protocol describes the process of consensus-building on how to ensure informed, accessible public engagement and to strengthen public trust in a climate of divisiveness and misinformation, disinformation, and malinformation using mRNA vaccines as a case study. This process will uncover social, political, and cultural tensions but also shortcomings in ample and transparent communication with various sectors of society. Considering the devastating effects that vaccine hesitancy has on population health, as seen recently in the case of measles resurgence in Canada, these dialogues will serve as a bridge between groups that are often disconnected or whose perspectives are portrayed as opposing. Overall, the study will cultivate collective capacity for dialogue on complex issues that affect our collective health, contributing to rebuilding healthy trust in public health institutions as they adapt to new societal demands.

## Supplementary material

10.2196/91534 Multimedia Appendix 1 Big-Tent criteria for excellent qualitative research.

10.2196/91534 Multimedia Appendix 2 Note-taking guide.

## Abbreviations

BRC: Bridge Research Consortium
CCGHR: Canadian Coalition for Global Health Research
CIEBH: Canada’s Immuno-Engineering and Biomanufacturing Hub
mRNA: messenger RNA
UBC: University of British Columbia
