# Supplementary material for: Healthy Trust and Pandemic Readiness in the Context of Messenger RNA Vaccines: Protocol for a Sequential Deliberative Dialogue Study
Source: JMIR Res Protoc. 2026 Sep 25;15:e91534. doi: 10.2196/91534 (PMC13614397; doi:10.2196/91534)
Supplement: Supplementary file 1 [file resprot-v15-e91534-s001.docx]

**Multimedia Appendix 1**

Eight “Big-Tent” Criteria for Excellent Qualitative Research^[1]^ – Examples of application in the manuscript

| **Criteria for quality** | **Description** | **Examples of application in the manuscript** |
| --- | --- | --- |
| Worthy topic | The topic of the research is   - Relevant - Timely - Significant - Interesting | The topic and focus of dialogue for this study was developed in direct response to questions raised in both the literature, in the media and in public discourses, and among people in leadership roles related to pandemic readiness. It focuses on drawing lessons and insights from the COVID-19 pandemic, as a means of adapting and preparing for future threats to collective health. Details of the relevance, timeliness, significance and interest in the topic are described in the **Introduction**  and **Discussion** sections. |
| Rich rigor | The study uses sufficient, abundant, appropriate, and complex   - Theoretical constructs - Data and time in the field - Sample(s) - Context(s) - Data collection and analysis processes | The study is heavily informed by theory (conceptualizations of trust) and so are the study design and methodology (grounded in critical pedagogy, critically reflective dialogue) as demonstrated in the **Methods** section, more specifically in the ***Study Setting & Sampling Strategy*** and ***Dialogue Process and Formats*** sub-sections. |
| Sincerity | The study is characterized by   - Self-reflexivity about subjective values, biases, and inclinations of the researcher(s) - Transparency about the methods and challenges | Reflexivity and transparency are inherent to the dialogic method, and the tangible plan to adhere to these practices is notably demonstrated in the ***Data Handling and Analysis*** sub-section. |
| Credibility | The research is marked by   - Thick description, concrete detail, explication of tacit (nontextual) knowledge, and showing rather than telling - Triangulation or crystallization - Multivocality - Member reflections | This criterion is not as relevant to a protocol since data is not reported, but attention to member-checking and commitment to representativeness of findings are described as central parts of the ***Dialogue Process and Formats*** and ***Data Handling and Analysis***. |
| Resonance | The research influences, affects, or moves particular readers or a variety of audiences through   - Aesthetic, evocative representation - Naturalistic generalizations - Transferable findings | Not applicable to the protocol as findings are not reported. |
| Significant contribution | The research provides a significant contribution   - Conceptually/theoretically - Practically - Morally - Methodologically - Heuristically | Mostly not applicable to the protocol as findings are not reported, however the outcomes of this work are intended to generate useful, practical, and applied directions for people across the spectrum of society whose work affects or is affected by pandemic readiness. Practical and moral contributions are largely considered in the **Introduction** and **Discussion** sections. |
| Ethical | The research considers   - Procedural ethics (such as human subjects) - Situational and culturally specific ethics - Relational ethics - Exiting ethics (leaving the scene and sharing the research) | Procedural ethics are reported in the ***Ethical Considerations sub-section***. Situational and culturally specific ethics, and relational ethics are considered throughout the **Methods** section. |
| Meaningful coherence | The study   - Achieves what it purports to be about - Uses methods and procedures that fit its stated goals - Meaningfully interconnects literature, research questions/foci, findings, and interpretations with each other | Not applicable to the protocol as findings are not reported; however, our analytical approaches and responsive study design are intended to achieve meaningful coherence, as described in the **Methods** section. |

1. Tracy, S. J., & Hinrichs, M. M. (2017). Big tent criteria for qualitative quality. *The international encyclopedia of communication research methods*, 1-10.
