# Supplementary material for: Healthy Trust and Pandemic Readiness in the Context of Messenger RNA Vaccines: Protocol for a Sequential Deliberative Dialogue Study
Source: JMIR Res Protoc. 2026 Sep 25;15:e91534. doi: 10.2196/91534 (PMC13614397; doi:10.2196/91534)
Supplement: Supplementary file 2 [file resprot-v15-e91534-s002.docx]

**Multimedia Appendix 2 - NOTE TAKING GUIDE AND TEMPLATE**

**Note: Chatham House Rule is in effect:** Anyone who comes to a meeting is free to use information from the discussion but is not allowed to reveal who made any comment.

The above means that when taking notes,

•	Attribution of comments to participants is not required

•	Listen closely for inputs that respond to our big questions

•	Listen closely for emergent themes – relevant to our project

•	Word-for-word transcription not required – we will turn on transcription

Role of Notetakers

Notetakers play an important role during the deliberative dialogues. You will help create a summary of dialogues and act as a listener and observer.

Please bring a charged laptop/tablet and charger with Microsoft Office Word for note taking. Please let us know if you do not have a device you can use so arrangements can be made for you to have access to one.

When taking notes

- Keep all information about the dialogues confidential and avoid using any personal identifiers (names or references toward individuals).
- Date and label which deliberative dialogue and small group (if applicable) you are recording notes for
- Write observations in bullet points and include sufficient detail so that the notes have meaning without additional explanation.
- If you use any shorthand while taking notes, please revisit to fully write each word out after the event.
- Organize notes clearly by emphasizing key points and indenting minor points and examples.
- Write the notes out verbatim (exactly how you hear them).
- Avoid inserting your opinion by focusing on what participants say, rather than your thoughts about what they say.

Record all**:**

- Key insights
- Powerful quotes or messages; and
- Consensus/validation (nodding, agreement, thumbs up)

After taking notes

Email completed notes to equityscience.lab@ubc.ca within 24 hours of the event and erase the file from your desktop once you have emailed the notes.

**Guiding Questions**

*These questions have been entered into template below to provide a way to organize notes.*

Have we got this right (synthesis presented during introduction)? What else would you add to further the understanding of the relationship between public sentiments, trust, and mRNA vaccines?

What would be useful to have come out of these dialogues?

Where does public health belong? What does it mean to expand public health?

Two recurring themes for action we've seen in dialogues are: 1) to broaden public health beyond what's thought of as official public health institutions and 2) to centre public health around communities. How can we work towards that?

General comments

*Enter notes here*

Questions for reflection and dialogue

**1. Have we got this right? What else would you add to further the understanding of the relationship between public sentiments, trust, and mRNA vaccines?**

*Enter notes here*

**2**. **What would be useful to have come out of these dialogues?**

*Enter notes here*

**3. Where does public health belong? What does it mean to expand public health?**

*Enter notes here*

**4**. **Two recurring themes for action we've seen in dialogues are: 1) to broaden public health beyond what's thought of as official public health institutions and 2) to centre public health around communities. How can we work towards that?**

*Enter notes here*

Emergent themes (enter a short title in each row, enter new rows as needed)

Key closing comments

*Enter notes here*
